# Chinese Medicine Shensongyangxin Is Effective for Patients with Bradycardia: Results of a Randomized, Double-Blind, Placebo-Controlled Multicenter Trial

**DOI:** 10.1155/2014/605714

**Published:** 2014-01-16

**Authors:** Yunfang Liu, Ning Li, Zhenhua Jia, Feng Lu, Jielin Pu

**Affiliations:** ^1^Department of Diagnosis, Shandong University Medical School, Jinan, Shandong 250014, China; ^2^Research Center for Pathology and Physiology, Cardiovascular Institute and Fu Wai Hospital, Peking Union Medical College, Chinese Academy of Medical Sciences, Beijing 100037, China; ^3^Integration of Traditional and Western Medical Research Academy of Hebei Province/Key Research Centre of Collateral Disease of State Administration of Traditional Chinese Medicine, Shijiazhuang, Hebei 050035, China; ^4^Affiliated Hospital of Shandong University of Traditional Chinese Medicine, Jinan, Shandong 250014, China

## Abstract

To evaluate the efficacy and safety of Shensong Yangxin (SSYX) in patients with bradycardia arrhythmias, a randomized, double-blind, and placebo-controlled study was conducted. Patients with bradycardia were randomly assigned to receive either SSYX (trial group, *n* = 115) or placebo (control group, *n* = 104) for 4 weeks. ECG, 24-hour continuous ECG recording, echocardiography, and hepatic and renal function were evaluated at baseline and after treatment. Results showed that the average heart rate, the fastest heart rate, and the lowest heart rate in the trial group were all significantly higher than those in the control group at the end of treatment (*P* < 0.05 or 0.01, resp.). Compared with pretreatment, the average heart rate, the fastest heart rate, and the lowest heart rate in the trial group all increased significantly after treatment (*P* < 0.05 or 0.01, resp.). Both the efficacy and the symptom scores in the trial group were significantly better than those in the control group after treatment (both having *P* < 0.01). No severe adverse effects were reported. In conclusion, SSYX treatment significantly increased the heart rate in patients with bradycardia without severe side effects. The exact mechanisms remain to be further explored.

## 1. Introduction 

Bradycardia is classified as a pulse rate below 60 beats per minute and is a common phenomenon in both healthy and disease conditions [1, 2]. Bradycardia may be a common manifestation of general conduction system disease or iatrogenic, due to medications used for atrial fibrillation rate control [3], which is characterized by a spectrum of arrhythmias including sinus bradycardia, sinus pauses, atrial fibrillation or flutter, and paroxysmal supraventricular tachycardia. The symptoms of bradyarrhythmia are most commonly intermittent syncope or presyncope but may be general and nonspecific. Some patients with sinus node disease are unable to appropriately increase their heart rate with exercise. Others may present with symptoms due to the underlying cause, such as myocardial infarction or drug toxicity.

Bradycardia can include sinus-node dysfunction (sick sinus syndrome) and atrioventricular block [4, 5]. The symptoms are often related to the bradycardia itself, as well as dysfunction of the autonomic nerve system (e.g., dizziness, fatigue, weakness, chest distress, or heart failure). In such cases, treatment targeted solely to correct bradycardia may not be effective in elimination of symptoms [6]. Besides, currently available drugs (e.g., atropine, isoproterenol, and theophylline) used to increase sinus rhythm are not tolerated for long term because of their side effects [7–9]. Since overdrive suppression of sinus automaticity may result in long pauses, syncope often occurs when tachycardia terminates [10]. Management for bradycardia-tachycardia syndrome is difficult because most antiarrhythmic drugs or the drugs that control the heart rate during tachycardia by blocking atrioventricular conduction such as *β*-adrenergic receptor blockers, calcium-channel blockers, or digitalis may lead to more severe bradycardia when the tachyarrhythmia terminates. For those patients, a cardiac pacemaker would usually be implanted before drug treatment [11]. Consequently, effective drugs with low side effects are of high interest as alternatives for treating those patients.

Some traditional Chinese medicines have been used to treat the disease related to arrhythmia for thousands of years. Shensongyangxin (SSYX) is a traditional Chinese medicine developed originally for treating cardiac tachyarrhythmias and it is a compound of the traditional Chinese materia medica consisting of 12 ingredients including *Panax ginseng*, dwarf lilyturf tuber, and Nardostachys root. Previous data suggested that SSYX has actions that may be beneficial in the treatment of symptomatic bradycardia. Small clinical studies demonstrated that SSYX effectively reduced ventricular premature beat and prevented bradycardia [12, 13]. Whole cell patch clamping experiments revealed that SSYX was a multiple ion channel blocker [14]. Preliminary studies suggested that SSYX can reduce the number of ventricular ectopic beats while mildly increasing the heart rate [15, 16]. However, extensive clinical trials have not been performed. Therefore, there are not evidence-based data from multicenter studies to confirm the clinical effects of SSYX. In order to further prove the effects of SSYX and cumulate the clinical data, a randomized, double-blind, placebo-controlled clinical study was designed to fully evaluate the efficacy and safety of SSYX in patients experiencing bradycardic arrhythmias.

## 2. Patients and Methods

### 2.1. Eligibility and Exclusion Criteria

This study was a randomized, double-blind, placebo-controlled, multicenter study. Patients with the age of 18 to 70 years accompanied by symptomatic bradycardia (average heart rate of 40~60 bpm) were enrolled to the study. However, patients indicated for permanent pacing therapy could be considered for the study only when the patients refused to do so.

Patients who met at least one of the following conditions were eligible: (1) sick-sinus syndrome with: *①* average ventricular rate of 40 to 50 bpm; *②* average ventricular rate of 50 to 60 bpm with the lowest rate less than 35 bpm accompanied by symptoms related to bradycardia; *③* sinus rhythm or other escape rhythms in atrium, AV junction, or ventricle that occurred ≥2.0 s after the termination of tachyarrhythmia; (2) atrioventricular block (AVB): *①* third degree AVB with documented asystole not longer than 2.5 seconds or escaped heart rate not slower than 40 bpm while awake; *②* second degree AVB; *③* first degree AVB with prolonged PR interval longer than 300 ms or with symptoms due to loss of atrioventricular synchrony; (3) chronic bifascicular and trifascicular block; (4) bradycardia-tachycardia syndrome.

Exclusion criteria included the following: (1) drug induced bradycardia; (2) endocrine or metabolism abnormalities and electrolyte disequilibrium; (3) acute myocardial infarction or unstable angina pectoris; (4) severe congestive heart failure (NYHA functional class III or IV); (5) uncontrolled hypertension; serious respiratory dysfunction or asthma; (6) liver, renal dysfunction; (7) primary hematopoietic system disease; (8) pregnant or child nursing; (9) allergy to study drugs; (10) patients enrolled to other drug studies; (11) blood pressure less than 90/60 mmHg; (12) bradycardia history shorter than 2 months; (13) syncope due to bradycardia; (14) R-R interval of ≥3.0 seconds; (15) patients who were currently treated with class I, III, or IV antiarrhythmic drugs were also excluded.

The study protocol was fully explained and written informed consent was obtained from each participant. The present study was approved by the Ethics Committee of Cardiovascular Institute and Fu Wai Hospital, Chinese Academy of Medical Sciences and Peking Union Medical College, and the Ethics Committees of the other 10 hospitals.

### 2.2. Study Design

This randomized, double-blind, placebo-controlled, multicenter trial was conducted at 11 hospitals across the mainland of China, during October 2007 to July 2008. Patients were randomized using Q-DAS statistical software by random permuted blocks and stratified by centers; the locations were divided into 11 regions, to orally receive either SSYX or placebo 1.6 g tid for 4 weeks. The SSYX capsule and placebo were produced and tested by Yiling Pharmaceutical Corporation (Shijiazhuang, China). They were identical in size, weight, color, and taste. Compliance with the study medication was monitored by counting the capsules individually.

Group assignment for all subjects was determined using a random table prior to the initiation of the study. The sequence of assignments was unknown to any of the investigators. Each assignment was kept in a sealed envelope and the order in numeric number was shown on the outside of the envelope. Thus, the orders could not be changed. Envelopes were arranged in order. The principal investigators generated this random selection a few months before recruiting the first subject. All the evaluations were performed by physicians or nurses who were blinded to the treatment given, using the same set of questionnaires and guidelines.

**Figure 1:**
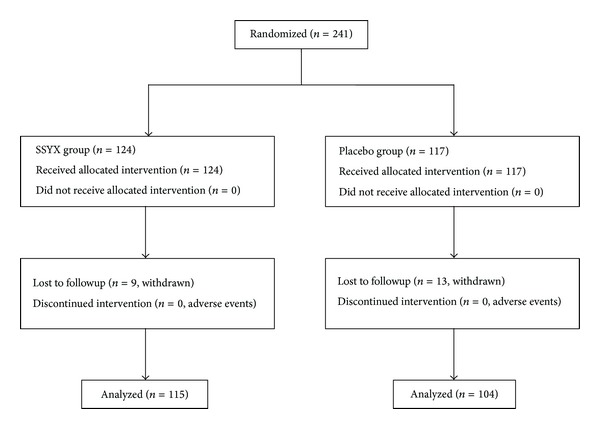
Participants screening and enrollment flowchart.

For all patients, the medical history, physical examination, blood tests (including serum glucose, electrolyte, GPT, GOT, creatinine, and urea nitrogen), 12-lead ECG, 24-hour Holter recording, and echocardiogram were screened. Diagnosis of the bradycardia was confirmed by 24-hour Holter recording. Follow-up clinical visits were scheduled at 4 weeks. All measurements were performed independently by two researchers and the values averaged. For all assays the intraobserver and interobserver variation coefficients were less than 5%, respectively. The participants screening and enrollment flowchart was shown in Figure 1.

The minimum sample size required for the study was calculated by the following. Compared with placebo, the experimental medicine curative effect is about 60%, control medicine is about 40%, alpha is 0.05, and beta is 0.2 (study power 80%), estimate for 98 cases in each group. Considering the drop factors, the study was designed to enroll 280 cases (140 cases in the trial group and 140 cases in the control group).

### 2.3. Outcome Measures

The primary outcome was objective criterion obtained by 24-hour Holter recording and the secondary outcome was subjective criterion obtained by symptom scores.

The criteria for assessing the therapeutic effects of SSYX capsules were formulated according to the “Guiding Principles of Clinical Research on Treatment of Coronary Heart Disease with Traditional Chinese New Drugs” published in 2002 [9] and the “Therapeutic Effects and ECG Evaluation Criteria for Treatment of Coronary Heart Disease” published in the Symposium for Coronary Heart Disease and Arrhythmia with integrated TCM and Western Medicine in 1998 [17].

The objective criteria for assessing the therapeutic effects of SSYX capsules on bradycardia were as follows according to the results obtained by 24-hour Holter recording. (1) A markedly effective response was defined as an increase of 20% or more in the average heart rate. (2) An effective response was defined as an increase from 10% to 20% in the average heart rate. (3) No effective response was defined as less than 10% increase or even decrease in the average heart rate.

The criteria for the symptom score depended on the frequency and degree of the symptoms which included palpitation, shortness of breath, fatigue, chest distress, agrypnia, and night sweat. (1) A markedly effective response was defined as the symptom scores decreased by >70%. (2) An effective response required a decrease from 30% to 70% in the symptom scores. (3) No effective response was defined as less than 30% decrease in the symptom scores.

### 2.4. Safety Monitoring

All patients underwent follow-up office visits weekly during therapy. At each follow-up, blood routine, transaminases, serum urine nitrogen (BUN), physical examination, echocardiography, and 12-lead ECG were measured and the incidence and severity of various side effects (i.e., nausea, vomiting, diarrhea, abdominal pain, headache, and dizziness) which may be associated with therapy were monitored.

### 2.5. Statistical Analysis

Continuous variables are given as mean ±SD. Comparison of the basic characteristics between the control group and the trial group was made using Student *t*-test. As the data were normally distributed, the difference between the two groups in terms of the changes of Holter recording before and after treatment was analyzed by repeated measures ANOVA. Pearson Chi-Square test or Fisher's exact test was used to compare categorical variables.

Calculations were performed with the statistical software SAS 9.1.3 (SAS Institute Inc., USA) and Q-DAS for Clinical Trial 3.0. (Q-DAS software technology, China). The SAS data sheets ITT (intention-to-treat was defined as patients who were treated at least once) and FAS (full analysis set was defined as the participants including eligible cases and drop-out cases) were used for efficacy and SS (safety set was defined as participants who received treatment and safety analysis at least once).

## 3. Results

### 3.1. Patient Characteristics

From October 2007 to July 2008, 241 consecutive patients were enrolled in this prospective study from 11 hospitals across the mainland of China. Of the 241 patients enrolled, 124 patients were randomized to the trial group and 117 patients to the control group. Nine patients in the trial group and 13 patients in the control groups were lost to follow up. As a result, a total of 219 patients (115 in the trial group and 104 in the control group) completed the study. The main patient characteristics and the types of bradycardia were summarized in Table 1. The arms of the study were well balanced with respect to age, gender, height, weight, blood pressure, average heart rate (AHR), fastest heart rate (FHR), lowest heart rate (LHR), left ventricular ejection fraction, QT interval, NYHA class, ischemic heart disease, history of bradycardia, sinus bradycardia, sinus pauses, atrioventricular block, and bradycardia-tachycardia.

### 3.2. Changes of Heart Rate

One patient in SSYX group and 4 patients in placebo group refused to repeat 24-hour Holter recording at the end of treatment.

As shown in Table 2, the average heart rate in the trial group was significantly higher than that in the control group after treatment (60.50 ± 8.70 versus 56.50 ± 5.99, *P* < 0.01). As for the effect of SSYX on heart rate, we found that the 24-hour average heart rate increased by 7.15 ± 7.43 in the trial group and by 2.60 ± 4.53 in the control group after treatment (*P* < 0.01). Both the fastest heart rate and the lowest heart rate in the trial group were significantly higher than those in the control group at the end of 4 weeks (*P* < 0.05 or 0.01, resp.). Compared with pretreatment, the average heart rate, the fastest heart rate, and the lowest heart rate in the trial group all increased significantly after treatment (*P* < 0.05 or 0.01, resp.). The average heart rate and the lowest heart rate in the control group were also increased significantly at the end of 4 weeks (*P* < 0.05 or 0.01, resp.), whereas the fastest heart rate did not change significantly (*P* > 0.05).

### 3.3. Objective Efficacy

The results of objective efficacy were analyzed in all of the participants including eligible cases and drop-out cases. As shown in Table 3, the markedly effective response rate was 24.2% in the trial group and 0.9% in the control group after treatment. The effective response rates were 34.7% in the trial group and 18.8% in the control group, respectively. Significant differences were found between the two groups (*P* < 0.01).

### 3.4. Symptom Scores

The results of symptom scores were shown in Table 4. Compared with the baseline level, the symptom scores in both groups decreased after treatment (3.42 ± 2.88% versus 7.66 ± 3.95 in the trial group and 4.97 ± 3.26% versus 7.35 ± 3.79 in the control group). Compared with the control group, the symptom score in the trial group was significantly lower (*P* < 0.01).

### 3.5. Adverse Events

Adverse events most possibly related to the treatment were reported in 1 (0.8%) patient in the trial group and 5 (4.3%) patients in the control group. Palpitation, headache, stomachache, and abdominal distension were the major symptoms. The symptoms completely recovered a few days after termination of the treatment. Neither death nor serious adverse event was reported during the study.

## 4. Discussion

The current study was designed to evaluate the efficacy and safety of SSYX in the treatment of patients with bradycardic arrhythmias. The results demonstrated that SSYX treatment significantly increased heart rate in patients with bradycardia. The total effective rate was 63.5% in the trial group and 22.1% in the control group. Symptom scores were also significantly improved in the trial group compared with the control group. These results compare well with studies that suggested that SSYX has effect on paroxysmal atrial fibrillation [18] and premature ventricular contractions [19].

The effects of SSYX in treating cardiac rate and contraction abnormalities may be conferred by such ingredients of SSYX as *Panax ginseng*, which may balance the tension of autonomic nerve system [20]. Our previous electrophysiological study demonstrated that SSYX increased heart rate and enhanced conducting capacity of the heart in the Chinese miniature swine only when the autonomic nervous system was intact [21]. Another research has shown that SSYX affects cardiac action potentials by lowering the L type calcium channel currents and transient outward potassium currents in rabbit pulmonary vein myocytes [22], while a study on guinea pig ventricular myocytes showed that these channels were blocked [16] and another study found that SSYX could block these and many other ion channels such as the sodium current, the inward rectifier potassium current, and the delayed rectifier current, in rat and guinea pig ventricular myocytes [14].

It is earlier to come to a conclusion on the effect of SSYX on patients with II (type 2) or III degrees of atrioventricular block because of the small number of patients tested. For the sake of safety, patients with extreme bradycardia (e.g., heart rate below 40 beats per minute while awake, R-R interval exceeding 3.0 seconds in sinus rhythm, or experiencing syncope probably caused by bradycardia) were excluded in the trial. For those patients, implantation of a pacemaker is recommended if necessary.

There was no death or severe adverse events registered during the study. No significant changes in hepatic and renal function have been observed immediately after study. Neither proarrhythmic events nor QTc prolongation related to SSYX treatment were recorded. The most common adverse effects were temporary stomachache and abdominal distension. The outcomes of the study suggested that clinical use of SSYX was safe and effective for treating patients with sinus bradycardia.

There are some limitations in our study. First, the observation time is only 4 weeks and hence a longterm study is obviously needed to further confirm our results. Second, clinical and laboratory findings were used as the endpoints in our study, whereas a hard clinical endpoint such as mortality should be used in a large sample of patients in a future study. Third, a positive control is not set in this study and we will evaluate if SSYX is optimal efficiency or noninferiority in the future.

In conclusion, this study shows that SSYX is safe and effective for the treatment of bradycardia. This result means that SSYX should be considered an alternative treatment for bradycardic patients, especially those who are not recommended for pacemakers or patients who choose not to have a pacemaker fitted.

## Figures and Tables

**Table 1:** Basic characteristics of study population.

Groups	Trial group (*n* = 115)	Control group (*n* = 104)
Gender M/F (*n*)	60/55	47/57
Height (cm)	165.68 ± 7.85	165.15 ± 7.71
Weight (kg)	66.69 ± 10.24	65.35 ± 9.99
Systolic blood pressure (mmHg)	126.64 ± 13.64	128.26 ± 13.70
Diastolic blood pressure (mmHg)	77.84 ± 7.71	76.51 ± 8.33
Heart rate (bpm)	54.09 ± 5.87	53.13 ± 5.79
QT interval (ms)	430.42 ± 35.85	437.07 ± 34.32
Left ventricular ejection fraction (%)	65.88 ± 6.68	65.28 ± 8.04
Left ventricular end diastolic dimension (mm)	49.70 ± 6.50	48.63 ± 5.57
NYHA class, *n* (%)		
I	95 (82.6)	86 (82.7)
II	20 (17.4)	18 (17.3)
Hypertension, *n* (%)	37 (32.2)	32 (30.8)
Ischemic heart disease, *n* (%)	32 (27.8)	39 (37.5)
Diabetes mellitus *n* (%)	6 (5.2)	7 (6.7)
History of bradycardia *n* (%)	13 (11.3)	7 (6.7)
Types of bradycardia (%)		
Sinus bradycardia, sinus pauses	64 (55.7)	65 (62.5)
Atrioventricular block	11 (9.6)	8 (7.7)
Bradycardia-tachycardia syndrome	40 (34.8)	31 (29.8)

**Table 2:** Heart rate in the trial group and the control group at baseline and 4 weeks.

HR variables	Trial group	Control group	*P* value
AHR (bpm)			
Baseline	53.38 ± 5.16	53.83 ± 4.17	0.482
4 weeks	60.50 ± 8.70*	56.50 ± 5.99*	0.000
FHR (bpm)			
Baseline	95.50 ± 24.94	95.31 ± 20.64	0.952
4 weeks	105.54 ± 24.97^#^	98.93 ± 18.85	0.029
LHR (bpm)			
Baseline	33.50 ± 3.62	33.98 ± 3.91	0.350
4 weeks	40.74 ± 7.67^#^	38.08 ± 5.85^#^	0.005

HR: heart rate; AHR: average heart rate; FHR: fastest heart rate (bpm); LHR: lowest heart rate.

At baseline, there are 115 patients in the trial group and 104 patients in the control group. At the end of 4 weeks, 1 patient in the trial group and 4 patients in the control group refused to repeat 24-hour Holter, so their HR variables are missed.

*P < 0.05 versus pretreatment in the same group.

^#^ *P* < 0.01 versus pretreatment in the same group.

**Table 3:** The objective efficacy comparison between the trial group and the control group.

Group	*n*	MER (%)	ER (%)	NER (%)	*P* value
Trial group	124	30 (24.2)	43 (34.7)	51 (41.1)	0.000
Control group	117	1 (0.9)	22 (18.8)	94 (80.3)	

MER: markedly effective response; ER: effective response; NER: no effective response.

**Table 4:** The symptom scores analysis between the trial group and the control group.

Time	Trial group (*n* = 115)	Control group (*n* = 104)	*P* value
0 week	7.66 ± 3.95	7.35 ± 3.79	0.547
4 weeks	3.42 ± 2.88	4.97 ± 3.26	0.000

## References

[1] Holly RG, Shaffrath JD, Amsterdam EA (1998). Electrocardiographic alterations associated with the hearts of athletes. *Sports Medicine*.

[2] Groh WJ (2012). Arrhythmias in the muscular dystrophies. *Heart Rhythm*.

[3] Feng L, Gong J, Jin Z-Y (2009). Electrophysiological effects of Chinese medicine Shen song Yang xin (SSYX) on Chinese miniature swine heart and isolated guinea pig ventricular myocytes. *Chinese Medical Journal*.

[4] Durham D, Worthley LI (2002). Cardiac arrhythmias: diagnosis and management. The bradycardias. *Critical Care and Resuscitation*.

[5] Ufberg JW, Clark JS (2006). Bradydysrhythmias and atrioventricular conduction blocks. *Emergency Medicine Clinics of North America*.

[6] Brady WJ, Harrigan RA (2001). Diagnosis and management of bradycardia and atrioventricular block associated with acute coronary ischemia. *Emergency Medicine Clinics of North America*.

[7] Das G (1989). Cardiac effects of atropine in man: an update. *International Journal of Clinical Pharmacology Therapy and Toxicology*.

[8] Hendeles L, Weinberger M, Milavetz G, Hill M, Vaughan L (1985). Food-induced “dose-dumping” from a once-a-day theophylline product as a cause of theophylline toxicity. *Chest*.

[9] Zipes DP, Camm AJ, Borggrefe M (2006). ACC/AHA/ESC 2006 guidelines for management of patients with ventricular arrhythmias and the prevention of sudden cardiac death: a report of the American College of Cardiology/American Heart Association Task Force and the European Society of Cardiology Committee for Practice Guidelines. *Journal of the American College of Cardiology*.

[10] Aronow WS (2009). Management of atrial fibrillation in the elderly. *Minerva Medica*.

[11] Rucinski P, Rubaj A, Kutarski A (2006). Pharmacotherapy changes following pacemaker implantation in patients with bradycardia-tachycardia syndrome. *Expert Opinion on Pharmacotherapy*.

[12] Huang JZ, Yan H, Chen XG, Liang JC (2006). Effect of Shensong Yangxin Capsule on ventricular premature beat and heart rate variablity. *Modern Medicine and Health*.

[13] Shen XX, Lu WX (2008). Effect of Shensong Yangxin Capsule on sinus node bradycardi. *Chinese Journal of Information on TCM*.

[14] Li N, Ma K-J, Wu X-F, Sun Q, Zhang Y-H, Pu J-L (2007). Effects of Chinese herbs on multiple ion channels in isolated ventricular myocytes. *Chinese Medical Journal*.

[15] Chai S, Wang S, Yao L, Wu A, Liu Y, Rao C (2009). Effect of shensongyangxin capsule on myocardial remodeling and ventricular fibrillation threshold value in rat with coronary artery ligation. *Zhongguo Zhongyao Zazhi*.

[16] Li N, Huo Y-P, Ma K-J, Sun Q, Pu J-J (2007). Effects of solution of dry powder of ShenSongYangXin capsule on sodium current and L-type calcium current in ventricular myocytes: experiment with guinea pig. *National Medical Journal of China*.

[17] Chen KJ, Liao JZ, Xiao ZX (1998). *Research on Cardiocerebrovascular Diseases*.

[18] Wang A-H, Pu J-L, Qi X-Y (2011). Evaluation of shensongyangxin capsules in the treatment of paroxysmal atrial fibrillation: a randomized, double-blind and controlled multicenter trial. *Zhonghua yi Xue Za Zhi*.

[19] Zou J-G, Zhang J, Jia Z-H, Cao K-J (2011). Evaluation of the traditional chinese medicine shensongyangxin capsule on treating premature ventricular contractions: a randomized, double-blind, controlled multicenter trial. *Chinese Medical Journal*.

[20] Zheng A, Moritani T (2008). Effect of the combination of ginseng, oriental bezoar and glycyrrhiza on autonomic nervous activity and immune system under mental arithmetic stress. *Journal of Nutritional Science and Vitaminology*.

[21] Essebag V, Hadjis T, Platt RW, Pilote L (2003). Amiodarone and the risk of bradyarrhythmia requiring permanent pacemaker in elderly patients with atrial fibrillation and prior myocardial infarction. *Journal of the American College of Cardiology*.

[22] Shi L, Yang X-C, Liu X-L, Zong M, Wu Y-L (2009). Effects of ShenSongYangXin on action potential and some current channels in isolated rabbit pulmonary vein cardiomyocytes. *Zhonghua yi Xue Za Zhi*.

